# Modelling the mechanoreceptor’s dynamic behaviour

**DOI:** 10.1111/joa.12328

**Published:** 2015-06-25

**Authors:** Zhuoyi Song, Robert W Banks, Guy S Bewick

**Affiliations:** 1 Centre for Mathematics, Physics and Engineering in the Life Sciences and Experimental Biology (CoMPLEX), University College LondonLondon, UK; 2 School of Biological and Biomedical Sciences, University of DurhamDurham, UK; 3 School of Medical Sciences, Institute of Medical Sciences, University of AberdeenAberdeen, UK

**Keywords:** biophysical model, fly photoreceptor, refractory period, sensory adaptation, sensory habituation, stochastic adaptive sampling, stretch-sensitive mechanoreceptor

## Abstract

All sensory receptors adapt, i.e. they constantly adjust their sensitivity to external stimuli to match the current demands of the natural environment. Electrophysiological responses of sensory receptors from widely different modalities seem to exhibit common features related to adaptation, and these features can be used to examine the underlying sensory transduction mechanisms. Among the principal senses, mechanosensation remains the least understood at the cellular level. To gain greater insights into mechanosensory signalling, we investigated if mechanosensation displayed adaptive dynamics that could be explained by similar biophysical mechanisms in other sensory modalities. To do this, we adapted a fly photoreceptor model to describe the primary transduction process for a stretch-sensitive mechanoreceptor, taking into account the viscoelastic properties of the accessory muscle fibres and the biophysical properties of known mechanosensitive channels (MSCs). The model’s output is in remarkable agreement with the electrical properties of a primary ending in an isolated decapsulated spindle; ramp-and-hold stretch evokes a characteristic pattern of potential change, consisting of a large dynamic depolarization during the ramp phase and a smaller static depolarization during the hold phase. The initial dynamic component is likely to be caused by a combination of the mechanical properties of the muscle fibres and a refractory state in the MSCs. Consistent with the literature, the current model predicts that the dynamic component is due to a rapid stress increase during the ramp. More novel predictions from the model are the mechanisms to explain the initial peak in the dynamic component. At the onset of the ramp, all MSCs are sensitive to external stimuli, but as they become refractory (inactivated), fewer MSCs are able to respond to the continuous stretch, causing a sharp decrease after the peak response. The same mechanism could contribute a faster component in the ‘sensory habituation’ of mechanoreceptors, in which a receptor responds more strongly to the first stimulus episode during repetitive stimulation.

## Introduction

Biological sensory receptors have to constantly adapt to effectively represent the great variation of input intensities in their intrinsically limited output range (van Hateren & van der Schaaf, 1996). Environmental stimuli can change over several orders of magnitude as diurnal animals navigate through their habitats,^1^ whereas sensory receptors can only change across tens of mV in their receptor potentials (Rieke & Rudd, 2009). The significant differences in the input and output ranges impose common engineering objectives on all sensory systems: how to effectively represent the vast input intensity changes within a limited output range so that faint signals are not buried in the noise, nor is the system completely saturated under intense stimuli (van Hateren & van der Schaaf, 1996; Rieke & Rudd, 2009).

Sensory receptors have evolved with sophisticated adaptive mechanisms to adjust their responses (Torre et al. 1995). Yet, there are no commonly accepted explanations of how various steps work together to produce the temporal dynamics to even the simplest step-like stimuli (De Palo et al. 2013), where adaptation could happen in multiple timescales (Wark et al. 2009). Part of the reason is because transduction cascades in different sensory modalities have notable differences in the molecular components and their reaction mechanisms, which add to the complexities in comparing biophysical mechanisms associated with sensory adaptation.

**Fig 1:**
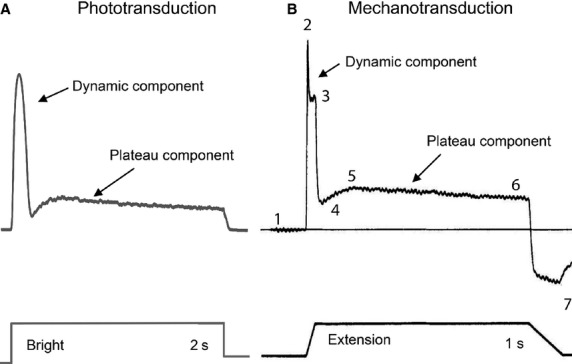
A photoreceptor and a mechanoreceptor exhibit remarkably similar response dynamics to step-like stimuli. (A) Light-induced-current in response to bright square pulse in a fly photoreceptor (reproduced from Song et al. 2012). A large initial peak quickly drops to a post-dynamic minimum, which then recovers to a much smaller plateau. The peak dynamic component is called fast adaptation, which takes only 200–500 ms before transition to the plateau. The exponential trend at plateau is slow adaptation. (B) In a primary ending of mammalian muscle spindle, a ramp-and-hold stretch evokes a comparable characteristic pattern of potential change, consisting of a large dynamic depolarization during the ramp phase and a smaller static depolarization during the hold phase (reproduced from Hunt et al. 1978). 1–7 are numbered in the same way as in fig.1 in Hunt et al. (1978), representing different components in the rich response dynamics. They are named as: (1) baseline; (2) peak of initial dynamic component; (3) peak of late dynamic; (4) post-dynamic minimum; (5) static maximum; (6) end static level; and (7) post-release minimum. Although for better comparison with the model outputs it is best to use patch-clamped recording of the stimuli-induced ionic flow, as there were no such data available in the literature for mechanotransduction, we showed sub-optimally the receptor potential of a primary ending of mammalian muscle spindle.

The aim of this paper is to use computational modelling approaches to investigate common adaptive mechanisms in sensory receptor cells across different modalities, more specifically, photoreceptors and stretch-sensitive mechanoreceptors. Despite the great differences in the physical stimuli these receptors are specialized to detect, a photoreceptor and a mechanoreceptor exhibit remarkably similar response dynamics to step-like stimuli (Fig.1). While this resemblance may be purely coincidental, here we investigate whether it may be explained by common underlying sensory mechanisms. In response to an intense bright square pulse, a fly photoreceptor produces a large initial peak in the light-induced current that quickly drops to a much smaller plateau, which then slowly adapts before settling to the steady-state (Hardie & Raghu, 2001). The rapid decrease in the peak component is called fast adaptation, in which the cell only takes 200–500 ms to transit to the following plateau (Fig.1A). The exponential decay during the plateau is called slow adaptation, which can take place continuously for 10–20 s (Juusola & Hardie, 2001). Similarly, in a primary ending of mammalian muscle spindle (Fig.1B), a ramp-and-hold stretch evokes a comparable characteristic pattern of potential change, consisting of a large dynamic depolarization during the ramp phase and a smaller static depolarization during the hold phase (Hunt et al. 1978). The remarkable similarity in the response dynamics from these receptor neurons motivates the question: do these cells employ generic biophysical mechanisms for their adaptation process? If so, how much do these generic mechanisms account for adaptation in the cell responses?

Understanding common and distinct mechanisms for adaptation in different senses is of great importance for the reverse engineering^2^ of these sensory systems, for example, to achieve modular designs in sensory prostheses. For greater predictability of the mechanisms underlying receptor responses, white-box biophysical models, which are assembled from adequate known knowledge of the relevant ion-channel kinetics, are preferable to descriptive kernel modelling approaches in system neuroscience. However, few such studies exist in the mechanosensory field due to the lack of knowledge of the transduction components in these nerve terminals (Chalfie, 2009).

This paper will show that a stochastic adaptive sampling mechanism, first developed in a phototransduction model, can explain a number of dynamic features in mechanosensory adaptation. We will first recap the stochastic adaptive sampling mechanism, which was obtained from opening a successful white-box biophysical model for the *Drosophila* phototransduction cascade (Song et al. 2012). Then we will adapt this visual system computational model to the equivalent counterpart for a mechanosensory terminal. Using the adapted model, we will show how stochastic adaptive sampling explains several dynamic features in a mechanosensory neuron’s response, namely, the initial peak component in the ramp-and-hold evoked response and the ‘sensory habituation’ phenomenon to repeated stimuli experiment.

## Model

### Fly photoreceptor model: a generic model based on a stochastic adaptive sampling approach

Combining *in vivo* single-cell electrophysiology with biophysical modelling, a very successful ‘white-box’ mathematical model was established to describe the input–output relationships of a fly photoreceptor (Song et al. 2012). The term ‘white-box’ means that the signalling pathway was modelled according to the known physiological stoichiometric and kinetic properties of individual components, so that the molecular reaction dynamics reproduce experimental results at every stage of verification. A ‘white-box’ model is constructed across the scales from molecular reactions through transduction cascades, and up to whole cell behaviour, so it is expected to replicate the cell’s final electrophysiological outputs with minimal parameter tuning.

Because of its valuable genetic toolbox, the *Drosophila* phototransduction cascade is so well studied that a wealth of knowledge is available for the transduction signalling pathways, making it an ideal starting point to assemble such a ‘white-box’ biophysical model (Song et al. 2012). The model not only correctly replicates the molecular dynamics for a single photon response, but can also predict the neuron’s macroscopic response over an enormous range (10^2^–10^7 ^photons s^−1^) and to time-series stimuli of variable statistics (Song et al. 2012; Song & Juusola, 2014).

Experimental evidence suggests a quantum mechanism operates for the detection of light. Most photoreceptors (if not all) transduce light quanta (photons of suitable energies) into unitary events, called elementary responses or ‘quantum bumps’ (Hecht et al. 1941; Fuortes & Yeandle, 1964; Henderson et al. 2000). The information processing in a photoreceptor depends on how photons in the light stimuli are sampled, how the sampled photons are transduced into electrical bumps and how these bumps are integrated together. To mimic such photon sampling and processing in the light-sensitive and light-insensitive parts of the real photoreceptor cells, the whole computational model linked the following four modules (Song et al. 2009, 2012): (i) a random photon absorption module, describing how incoming photons are distributed across a large population of sampling units (microvilli); (ii) a stochastic bump module, describing how photons are transduced into electrical bumps in each microvillus; (iii) a bump summation module, summing bumps from all microvilli; and (iv) a Hodgkin–Huxley module of the photoreceptor plasma membrane, converting the light-induced current to the cell’s voltage response (electrophysiological output).

The details of the phototransduction reactions and how they are mathematically modelled are not the focus of this paper, but what is important is the systematic view of signal transduction, obtained from opening the ‘white-box’. The conceptual understanding of the signal mapping can be designed into an algorithm with the following heuristic rules: a huge population of microvilli sample the incoming photons according to a Poisson distribution; photons are transduced into bumps inside single microvilli through stochastic reactions; a photon leads to a bump if the microvillus is not in its refractory state, otherwise the photon energy is lost; all bumps from all microvilli sum up the macroscopic light-induced current; the bump rate increases with stimulus photon rate (light intensity), but is constrained by structural limits (number of microvilli) and the length of the refractory period; adaptation is achieved by either bump adaptation (bumps shrink with increasing light levels) or quantum efficiency reduction (bump/photon ratio reduces). These heuristic rules were composed into an adaptive mechanism, which was termed the stochastic adaptive sampling mechanism (Song et al. 2012).

The stochastic adaptive sampling mechanism is a dual multi-scale counterpart to the underlying biophysical ‘white-box’. This approach is very useful because of its elegant simplicity and powerful predictability. In the case of the photoreceptor, it reduces the underlying signal transduction mechanisms to only four general factors: the size of the quantal events; their latency distributions; their refractory period distributions; and the number of transduction units. In the current article, we show that these four factors can easily be adapted to model mechanosensory transduction, where they will correspond, respectively, to a single mechanosensory ion-channel’s response, the ion-channels’ activation probabilities, the ion-channels’ inactivation probabilities, and the number of channels per nerve terminal.

The relative contributions of these four factors are determined through balanced positive and negative feedback interactions in molecular reaction pathways, which can be abstracted from a modelling point of view. The system’s behaviour can be predicted as long as the statistics of the four general factors are known or can be measured. In the next section, we will describe an adaptation of this generic model for mechanosensory receptors. In the Results, we will illustrate the applicability of the generic model to both a slowly adapting crayfish stretch receptor and a spindle mechanosensory primary ending.

### A generic biophysical model for mechanosensory primary ending

**Fig 2:**
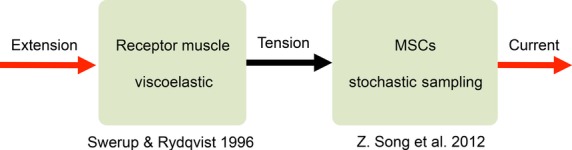
A simplified feed-forward model for mechanotransduction. The extension stimulus exerts tension onto the receptor muscle fibres, described by a viscoelastic model (adapted from Swerup & Rydqvist, 1996). The tension is then transduced into a receptor current by stochastic sampling of a large population of mechanosensitive channels (MSCs).

Mechanotransduction is the process by which mechanosensory cells detect physical stimuli, such as tension, stretch or pressure, and convert them into electrical responses within the nervous system (Chalfie, 2009). Primary mechanotransductions occur in specialized mechanosensory endings, and they share common processes (French, 1992). A simplified feed-forward pathway for mechanotransduction is conventionally viewed as a three-stage process (French, 1992): (a) the stimulus is mechanically coupled to the receptor cell, causing a deformation of the cell’s sensory terminal; (b) the deformation is transduced into an electrical signal (receptor current or potential) – the common view is that stretch-sensitive ion-channels within the endings are directly gated by mechanical stimuli; (c) the receptor potential is then encoded into action potentials for transmission to the nervous system. Here, we will describe a generic feed-forward biophysical model for processes (a) and (b) (depicted in Fig.2), but (c) is beyond the focus of this study. We will tailor the model to reproduce the ramp-and-hold extension-evoked receptor potential dynamics of stretch-sensitive mechanoreceptors, for example, a crayfish slow stretch receptor and a mammalian spindle primary ending. However, the beauty of this approach is that the modelling structure would not lose its generality in describing other mechanotransduction processes.

#### Viscoelastic model for receptor muscle components

Mechanosensory endings may implement diverse structures to couple mechanical stimuli to the deformation of their dendrite membranes. For example, mammalian spindles directly incorporate the termini of stretch-sensitive afferent neurons. Their sensory terminals in turn adhere to the surface of the intrafusal muscle fibres (Bewick & Banks, 2015). Direct observation of isolated or semi-isolated muscle spindles shows that stretch of the spindle is accompanied by extension of the sensory region and measureable increase in the spacing between the turns of the primary-ending terminals (Boyd, 1976; Poppele & Quick, 1985).

**Fig 3:**
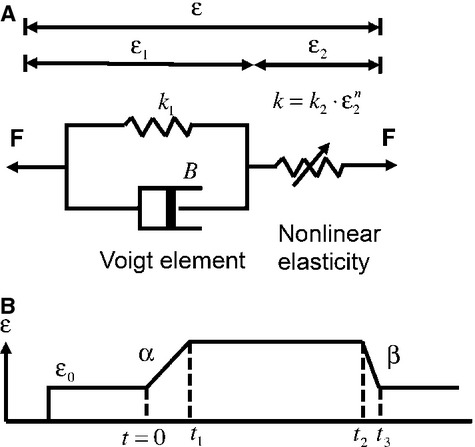
(A) Viscoelastic model used to represent receptor muscle (Swerup & Rydqvist, 1996). Total extension is the sum of that from both linear (left-hand-side spring in A) and non-linear springs (right-hand-side spring in A). The spring constant of the non-linear spring is *k*  = *k*_2_ × *ε*^*n*^. (B) General form of the ramp-and-hold extension. *ε*_0_ is extension before ramp, *t*_1_ is the end of extension rising phase, *α* is rate of rise, *t*_2_ and *t*_3_ define the falling phase of the ramp, *β* is the rate of fall.

Studying the mechanical properties of the associated muscle fibres is crucial in understanding how receptor muscle length changes (stretch stimuli) are mapped to the tension changes on mechanosensory endings. These tension changes are closely related to the forces or pressure on the sensory terminals, composing the gating forces to the mechanosensitive channels (MSCs; Chalfie, 2009). Although both direct experimental measurement (Hunt & Wilkinson, 1980) and theoretical estimate (see Banks in this volume) of steady-state tension of a cat muscle spindle gives a range of tension changes, no direct measurement of the tension temporal profiles has been made. Therefore, tension profiles normally rely on approximations from viscoelastic models. A simple form of the models was proposed in the 1960s to describe the adaptive viscoelastic properties of the intrafusal muscle fibres (Matthews, 1964). The model has two components connected in series: one Voigt component consists of elasticity (a spring) and viscosity (a dash-pot) in parallel; and the other component consists of a pure elasticity (Fig.3A, reproduced from fig.1A in Swerup & Rydqvist, 1996). The dendrite terminals are supposed to be attached to the pure elasticity on the right-hand side (Fig.3A). More recently, the same model structure was used to describe the adaptation in the tension profiles of *Astacus astacus* stretch receptor muscle, which show strong analogy to mammalian muscle spindles (Swerup & Rydqvist, 1996; Suslak et al. 2011). This illustrates the wide applicability of the viscoelastic model for describing the dynamic behaviours of receptor muscles. To give the model more parameter-fitting freedoms, the pure elastic spring on the right-hand side was changed to a non-linear spring in these recent works (Fig.3A). We will next re-implement this updated model and slightly modify it when characterizing the possible tension changes for a mammalian muscle spindle primary ending.

The stimuli for the viscoelastic model are ramp-and-hold extensions because of their extensive usage in mechanosensory experimental work (Fig.3B). Principally, the receptor muscle tension (*σ*_m_) varies with the extension (*ε*), given by: 

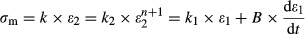
1

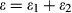
2where *k* is the spring constant of the non-linear spring, and it is an exponential function of the non-linear spring’s extension: 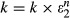; *k*_1_ is the spring constant of the left-hand spring in Fig.3A, in parallel of which is a dashpot with a viscous constant, *B*; *ε*_1_ is the extension of the left-hand spring (Fig.3A); the total extension of the muscle (*ε*) is the sum of *ε*_1_ and *ε*_2_; extensions are given as the percentage of initial muscle length.

Combining Eqs 1 and 2, a differential equation for *ε*_2_ can be obtained:

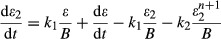
3where *ε*_2_ can be solved using the Runge–Kutta method.

To replicate the super-sensitivity of tension during the ramp phase, we introduced an adaptive ramp amplification factor *r* for the non-linear spring when it is responding to ramp stimuli: 

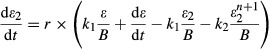
4

This factor was not implemented in the model described in Swerup & Rydqvist (1996), but it seems to be necessary here, so that a richer initial dynamical component can be introduced in the responses (see Results, ‘Initial peak component for fast adaptation in a mamalian muscle spindle’).

*ε*_2_ in Eqs 3 and 4 was then used to calculate the tension experienced by the receptor muscle (*σ*_m_) according to Eq. 1. The tension in the primary ending terminal membrane (*σ*), where MSCs are localized in high concentration, was assumed to be directly proportional to *σ*_m_: *σ* = *σ*_m_/*m*, where *m* is a constant.

#### Stochastic adaptive sampling from a huge population of MSCs

The open probability for the MSCs (*P*_0_) is a function of the tension in the primary ending terminal membrane, *σ*. The steady-state relationship between *P*_0_ and *σ* was calculated using a Boltzmann function: 

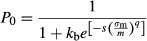
5where *k*_b_ is a constant, *s* is a sensitivity constant and the power constant *q* is 1 in this study. Eq. 5 is the typical way of mapping *σ*_m_ to *P*_0_ (Guharay & Sachs, 1984; Erxleben, 1989; Swerup & Rydqvist, 1996). However, crucially, it ignores the dynamical relationship of *P*_0_ to *σ*. Thus, it precludes the introduction of any extra adaptive dynamics from the MSC openings, if present, and as predicted in this model.

Here, we introduced a way to implement such dynamics into the computational model out of stochastic adaptive sampling, as is present in the fly phototransduction. Although mechanotransduction and fly phototransduction share great similarities in response dynamics, the detailed underlying reaction pathways responsible for these dynamics seem certain to deviate greatly. The fly phototransduction process uses a second-messenger reaction pathway to transduce the energy of a photon indirectly to Transient Receptor Potential channel openings. But, mechanosensory systems are generally thought to use direct transduction, which does not involve a chemical intermediate. Therefore, many properties of the intermediate second-messenger molecules in the fly phototransduction cascade could be collapsed into the equivalent activation and inactivation profiles of the MSCs.

Quantitative analysis of single MSC records reveals that the sensitivity to stretch can be described by a linear four-state model with one open (*O*) and three closed (*C*) states (Sachs, 1986). Here, for simplicity, we classify the closed states into two categories. One category is a refractory state (*R*), where the channel is inactivated and cannot be opened again, even though there is a stimulus. The other category is an available state (*A*), which means that the channel is available to be opened by an external stimulus. For this model, the sequence of channel activation is illustrated as follows: 

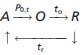
6

The number of newly opened MSCs at each moment in time is then calculated as: 

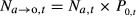
7where *N*_*a* →0,*t*_ is the number of newly opened channels at *t*, *N*_*a,t*_ is the number of available channels (closed channels that are not in their refractory period) at *t*, *P*_0,*t*_, is the channel open probability at *t*, calculated by Eq. 5. *N*_*a*,0_ is initialized as *N*_T_. All opened channels change into state *R* after *t*_o_, where *t*_o_ follows a uniform distribution between 1 and *t*_open_. All channels in state *R* will change to state A after *t*_r_, where *t*_r_ follows a uniform distribution between 1 and *t*_R_.

The macroscopic stretch-induced current, or receptor current (*I*_s_), is generated by the ionic flow through all the simultaneously opened individual MSCs: 

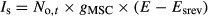
8where *E* is the membrane potential and *E*_srev_ is the reversal potential for the MSCs. In actual patch-clamp experiments, *E* is typically voltage-clamped to the cell’s resting membrane potential. *g*_MSC_ is the single MSC conductance.

The iterative implementation of Eqs 6,7,8 over time is performed according to stochastic sampling principles: at each moment, the channels that are opened are stochastically sampled from the available pool, which are indexed for a time period of *t*_o_ + *t*_r_, before they return back to the available pool again. Furthermore, *t*_o_ and *t*_r_ are determined according to their own distribution, respectively. To keep the model simple, these distributions are assumed to be uniform.

Logically, in order to transpose the phototransduction model directly into that for mechanotransduction, it is necessary to imagine digitized sampling of a continuous input (tension changes). This is harder to conceptualize than samples of discrete photons. However, as the mechanosensory response results from direct gating of a population of transduction channels, each with a unitary all-or-none current, the number of opening channels can be viewed as the samples of the continuous tension changes. In other words, an open channel is a discrete sample of the input at a quantum level. It is this way of thinking that forms the bridge between mechanotransduction and phototransduction within the stochastic adaptive sampling framework, as the commonality is how the number of discrete samples changes to specific stimuli of varying intensity.

## Results and discussion

### Simulation of a slowly adapting mechanoreceptor

**Fig 4:**
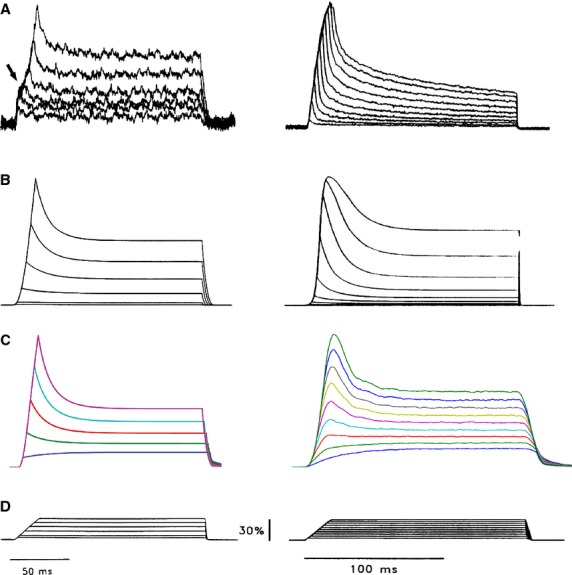
Tension (left panel), tension-induced-current (right panel) responses to ramp-and-hold extensions of a crayfish slowly adapting stretch receptor, and the model simulation outputs. (A) Recorded responses from a slowly adapting stretch receptor in response to ramp-and-hold extensions (1500% s^−1^) of 3–30% of muscle length (D). (B) Model-simulated responses (Swerup & Rydqvist, 1996). (C) Model-simulated responses (present model). An adaptive ramp amplification factor was added to the model of Swerup & Rydqvist (1996) to produce the tension profiles in (C, left). Stochastic sampling of MSCs was implemented to produce the tension-induced-current profiles (C, right). MSC opening duration, latency and refractory period were all uniformly distributed, with their respective maximum values listed in Table1. Interestingly, compared with channel opening times and latencies, refractory periods are much shorter. In fact, a ‘0 ms’ refractory period can produce equally good results (data not shown), indicating that a two-MSC state model (open and closed) can already take account of the slowly-adapting stretch receptor’s response dynamics. Other parameters used are listed in Table1. (D) Ramp-and-hold extension stimuli. Results are normalized, as the focus of the paper is not to fine-tune the parameters to reproduce the response absolute amplitude values, but only the temporal dynamics.

To verify this modelling framework for a mechanoreceptor, we first used the model to reproduce the ramp-and-hold extension-evoked responses of the crayfish slowly adapting stretch receptor. Crayfish stretch receptors are well studied, making them the prime choice for testing mechanosensory models. Experimental measurements of both receptor muscle tensions and the corresponding receptor potentials have been published (Rydqvist et al. 1990; Rydqvist & Swerup, 1991), and computational models have also been developed to map the receptor’s input–output relationships (Swerup & Rydqvist, 1996; Suslak et al. 2011). Thus, simulated results from the current modelling framework can be compared against both experimental measurements and simulation results from previous models. Figure4 shows such model validation results, where the left panel in the figure corresponds to tension profiles experienced by the muscle and the right panel displays the tension-induced-current profiles from the receptor sensory terminals. The parameters used for these simulations are listed in Table1.

**Table 1:** Model parameter values for Fig.4

Parameter	Description	Value (unit)	Sources
Viscoelastic elements			
*k*_1_	Spring constant for linear spring	200 (kPa)	SR96*
*k*_2_	Spring constant for non-linear spring	1100 (kPa)	SR96
*n*	Power constant for non-linear spring	1.2	SR96
*B*	Dashpot constant	12 (kPa)	SR96
*r*	Ramp amplification for non-linear spring	2	tuned
MSCs			
*k*_b_	Boltzmann constant (linear)	106	SR96
*S*	Sensitivity constant (linear)	0.00277 (Pa^−1^)	SR96
*Q*	Power constant (linear)	1	SR96
*M*	Tension conversion factor	80	Tuned
*g*_MSC_	Maximum unit conductance for the MSCs	35 (pS)	Tuned
*E*	Voltage-clamp potential	−70 (mV)	SR96
*E*_srev_	Reversal potential for MSC	+10 (mV)	SR96
*t*_open_	Maximum MSC opening time	10 (ms)	Tuned
*t*_1_	Maximum MSC response latency	10 (ms)	Tuned
*t*_r_	Maximum MSC refractory time	5 (ms)	Tuned
*N*_T_	Total number of MSCs	300 000	Tuned

* SR96 = Swerup & Rydqvist (1996). MSC, mechanosensitive channel.

In accordance with the actual experimental stimuli, ramp-and-hold extensions from 3 to 30% (Fig.4D) were applied to the present model (Fig.2) and to the previous model (Swerup & Rydqvist, 1996). Compared with experimental recordings, the present model simulations are comparable to that from the approach of Swerup & Rydqvist (1996). The present model produced tensions that have the same dynamics as that produced from the viscoelastic model in Swerup & Rydqvist (1996); indicating a correct re-implementation of their model. And vice versa, the current model deviates from experimental recorded tensions in the same way as shown in Swerup & Rydqvist (1996). One characteristic difference is that experimental data show a relatively larger stiffness in the low extension range, resulting in a typical ‘hump’ in the rising phase of the peak tension response (black arrow in Fig.4A, left).

Stochastic sampling of MSCs was implemented to produce the tension-induced-current profiles (Fig.4C, right). Although the present model simulations still deviate from experimental recordings (Fig.4A, right), they produce briefer and better peak dynamics than that shown in Swerup & Rydqvist (1996). In the modelling framework, MSC open times, latencies and refractory periods were all uniformly distributed, with their respective maximum values listed in Table1.

Interestingly, refractory periods are much shorter than channel open times and latencies. In fact, if the refractory period is reduced to 0 ms, equally good results are produced (data not shown), indicating that a more simplified two-MSC state model (open and closed) can readily account for this slow stretch receptor’s response dynamics.

### Initial peak component for fast adaptation in a mamalian muscle spindle

**Fig 5:**
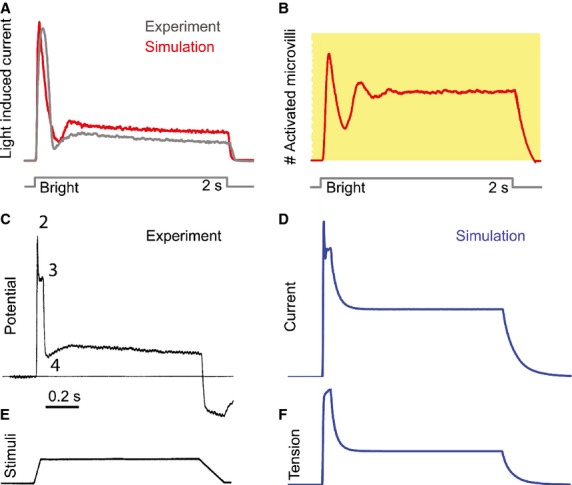
Stochastic sampling from a population of refractory units produces fast adaptation dynamics in a fly photoreceptor’s light-induced current (A and B) and initial peak dynamic component in a mammalian muscle spindle’s tension-induced-current (D). In a fly photoreceptor, at the onset of a bright light stimulus, all microvilli are sensitive to respond, inducing a sharp increase in the response. But as the microvilli become refractory, fewer and fewer are available to sample the next coming photons, resulting in a sharp decrease in the number of activated microvilli (B), and hence a fast adaptation dynamics in the light-induced current response (A). In a mammalian muscle spindle primary ending, ramp-and-hold extension stimulus (E) evokes rich dynamics in the receptor potential (C). Although for better comparison with the model outputs it is best to use patch-clamped recording of the stimuli-induced ionic flow, as there were no such data available in the literature for mechanotransduction, we showed the receptor potential as a sub-optimal substitute in (C). With such a comparison, at least one can see the dynamic components in the receptor potential that can already be produced with the stochastic adaptive sampling framework. The multiple dynamical components in the response are likely the combined results of biophysical mechanisms from different sources. A large adaptive ramp amplification factor (*r *= 10) is needed to produce the dynamic component 3 in (C), characterized by a small plateau on top of the tension profile (the small plateau in f replicates the dynamic component 3 in C). Otherwise, the tension profiles would look like that shown in Fig.4C, left, i.e. sharp peaks are produced without the small plateau. Unlike the fly photoreceptor microvilli, the refractory period of MSCs in a mammalian muscle spindle is much shorter, compared with its own open time. As a result, stochastic sampling from a population of refractory MSCs introduces the extra peak of the initial dynamic component (D replicates component 2 in C). In comparison, the stochastic sampling from refractory microvilli in a fly photoreceptor produces the post-dynamic minimum. In this particular simulation, the post-dynamic minimum (component 4 in C) is not produced in the tension-induced-current response.

In a mammalian muscle spindle, a primary ending responds to the simplest ramp-and-hold extension stimuli (Fig.5E) with considerably rich temporal dynamics, including initial peak dynamics (component 2 in Fig.5C), late peak dynamics (component 3 in Fig.5C), and post-dynamic minimum (component 4 in Fig.5C). The question is how to reverse engineer (i.e. design an equivalent) a system that can reproduce such rich response dynamics. We present here some scientific insights gained from simulating the stochastic adaptive sampling model for MSCs in a mammalian muscle spindle. The parameters for the model simulations in this section are listed in Table2.

**Table 2:** Model parameter values for Fig.5

Parameter	Description	Value (unit)	Sources
Viscoelastic elements			
*k*_1_	Spring constant for linear spring	100 (kPa)	SR96
*k*_2_	Spring constant for non-linear spring	2200 (kPa)	SR96
*n*	Power constant for non-linear spring	1.5	SR96
*B*	Dashpot constant	40 (kPa)	SR96
*r*	Ramp amplification for non-linear spring	10	Tuned
*MSCs*			
*K*_b_	Linear constant	10	Tuned
*m*	Tension conversion factor	300	Tuned
*g*_MSC_	Maximum unit conductance for the MSCs	35 (pS)	Tuned
*E*	Voltage-clamp potential	−70 (mV)	SR96
*E*_srev_	Reversal potential for MSC	+10 (mV)	SR96
*t*_open_	MSC opening time (fixed)	2 (ms)	Tuned
*t*_1_	MSC response latency (fixed)	0 (ms)	Tuned
*t*_r_	Maximum MSC refractory time	12 (ms)	Tuned
*N*_T_	Total number of MSCs	100 000	Tuned

MSC, mechanosensitive channel.

The multi-dynamical components are likely the combined results of both mechanical and ionic mechanisms (Grigg, 1986). The peak component is characterized by a dramatic drop following the end of the length increase. It was shown in a previous mechanical model for a muscle spindle that this drop, also called the dynamic index, is proportional to the velocity of stretch (Matthews, 1964). To reproduce this effect, a large adaptive ramp amplification factor (*r *= 10) is needed to produce the dynamic component 3 in Fig.5C, characterized by a small plateau on top of the initial peak of the tension profiles (Fig.5F). Otherwise, the tension profiles would just produce simple sharp peaks without the small plateau (Fig.4C, left). There is no initial dynamic component in the tension profiles (component 2 in Fig.5C is missing in Fig.5F) if no ionic mechanisms are included.

Just as fast adaptation dynamics emerge out of stochastic sampling from a huge population of refractory microvilli in photoreceptors (Fig.5A,B), the initial peak component in a primary ending’s receptor potential can be obtained by stochastic sampling from a population of refractory MSCs (Fig.5C,D). In a fly photoreceptor, at the onset of a bright light stimulus, all microvilli are sensitive to the stimulus, inducing the large initial increase in the response current. But as the microvilli become refractory, fewer and fewer of them are available to sample the subsequent incoming photons, resulting in a sharp decrease in the number of activated microvilli (Fig.5B), and producing the fast adaptation dynamics in the light-induced current response (Fig.5A). Similarly, stochastic sampling from refractory MSCs introduces the extra sharp peak in the dynamic component (Fig.5D replicating dynamical component 2 in Fig.5C). At the onset of a stretch stimulus, all MSCs are responsive. But, as the increased tension is maintained, they become refractory, and fewer are left available. This results in the initial rapid fall in receptor current (initial peak dynamical component 2 in Fig.5C). Thus, a prediction of the model is that the common mechanism that may underlie both the mechanoreceptor and a fly photoreceptor responses is that their sampling units (MSCs or microvilli) are both refractory in nature.

In contrast to the MSCs in a crayfish slow stretch receptor, which have very short refractory periods relative to their open time, the MSCs in a muscle spindle have much longer refractory periods than their open time. For example, to reproduce the initial peak dynamics of ramp-and-hold extension-evoked responses of a muscle spindle, the ratio between MSC refractory period to MSC open time (*t*_r_/*t*_o _= 6) is sixfold larger than that in a slow stretch receptor (*t*_r_/*t*_o _= 1). This indicates that a crayfish slow stretch receptor and a muscle spindle would have different groups of MSCs with distinct channel properties.

In preliminary studies of a more extreme simulation, it was found that an even longer refractory period (e.g. 120 ms, *t*_r_/*t*_o _= 60) is needed to produce the post-dynamic minimum (component 4 in Fig.5C, simulation data not shown). This infers that the initial peak dynamics and the post-dynamic minimum may result from mechanisms that span very different timescales. Of course, from a system point of view, similar response characteristics in the post-dynamic minimum could result from a differentiator, for which the mechanic solutions can be many. For example, the system may combine slow and fast populations of MSCs, or may have a single population of MSCs with multiple refractory states. Other equally valid mechanisms have also been suggested, such as current shunting by voltage-gated potassium conductances (Hunt et al. 1978). These possibilities will be explored in future developments of the model.

Although recent experimental evidence suggests that various feedback pathways can act as gain control mechanisms between the input and output of a primary sensory ending (Bewick & Banks, 2015), this model shows here that some adaptation phenomena can be accounted for without such feedback pathways. Certainly, the refractory nature of MSCs may be related to channel inactivation mechanisms, which may involve molecular reaction or ionic feedback pathways. Although the modelling framework presented here cannot unequivocally elucidate the detailed molecular mechanisms, it is useful for quantifying the necessary time constants of MSC activation and inactivation dynamics. This may provide valuable clues for screening MSC candidates in these mechanosensory systems.

### ‘Sensory habituation’ for repetitive stimuli

‘Sensory habituation’ describes the decline in responses of a primary sensory unit to a repetitive train of identical stimuli. The mechanisms underlying ‘sensory habituation’ are still mysterious. In a mechanosensory neuron, it may involve processes working on multiple timescales, including combined changes in the underlying graded potentials and in the spike induction processes (Pasztor & Bush, 1983).

**Fig 6:**
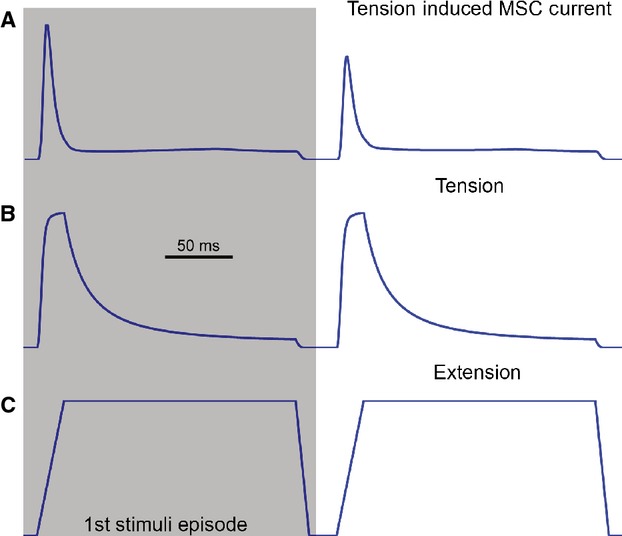
Stochastic sampling from a population of refractory mechanosensitive channels (MSCs) can produce ‘sensory habituation’ in a mechanoreceptor’s tension-induced-current profile, i.e. the first episode of response is larger than the second (A). Two episodes of the same extension pattern were applied sequentially to stimulate the modelled muscle spindle (C), evoking two episodes of same tension responses (B). However, the first episode of tension-induced-current response is larger than the second. The mechanisms underlying this ‘sensory habituation’ are still a mystery. Here, stochastic sampling from a population of refractory MSCs can reproduce to some extent the ‘sensory habituation’ effect. The reason is because while many MSCs are still refractory from responding to the first episode of stimulus, fewer MSCs are left to respond to the second episode of stimulus.

The present computational study shows that stochastic sampling from a population of refractory MSCs may contribute a faster component in the ‘sensory habituation’ of mechanoreceptors. Figure6 shows simulated results, as it has not yet been possible to undertake experimental validation of the simulations. This ‘playing’ with the model is an exercise in exploring the qualitative possibilities expected in experiments, which can be tested when exact quantification is carried out.

When stimulated with two identical episodes of ramp-and-hold extension stimuli (30%, with a ramp rate of 1500% s^−1^ in Fig.6C), the tension-induced-current of a mechanosensory ending decreases with the second stimulus (Fig.6A). As two identical episodes of tension responses were evoked in the simulations (Fig.6B), the ‘sensory habituation’ in the tension-induced-current responses must be caused by the MSC opening dynamics. In fact, to produce the response decline in the second episode of response, a relative long refractory period (it is extended 10-fold to 120 ms from 12 ms in the muscle spindles simulation, as shown in Table2) has to be used in the simulations. With this longer refractory period, fewer MSCs are left to respond to the second stimulus, as many MSCs are still refractory from the first.

Although adaptive voltage-dependent conductance changes can also produce such ‘sensory habituation’ phenomenon, stochastic adaptive sampling from refractory MSCs provides an alternative, and potentially simpler, explanation that might be explored experimentally. Indeed, stochastic adaptive sampling also explains ‘sensory habituation’ in fly photoreceptors in the same way (data not shown). Whatever the underlying system is being modelled, whether a fly photoreceptor or a mechanoreceptor, in the stochastic adaptive sampling framework, this ‘sensory habituation’ effect would be dependent on the number of refractory units, the distribution of their refractory periods, the duration of each stimulus and the interval between the two consecutive stimuli.

### Benefits of the stochastic modelling framework

There are some major advantages to this new stochastic modelling approach. Traditionally, the open probability for MSCs (*P*_0_) has been modelled as a non-linear equation of terminal membrane tension *σ*, for example, Boltzmann relationship in Guharay & Sachs (1984), Erxleben (1989) and Swerup & Rydqvist (1996). However, such calculations only consider the steady-state relationship between *P*_0_ and *σ*, but ignore the temporal dynamics of *P*_0_ subject to changes in *σ*. In the end, *P*_0_ would only be a non-linear static mapping of the temporal profile of *σ*. This static mapping cannot replicate the initial peak dynamic component in Fig.5C, which requires extra biophysical mechanisms, such as that in channel openings.

Another traditional approach is to use ordinary differential equations (ODEs) to model the relationships between *P*_0_ and *σ*, so that dynamics from a differentiator can be introduced. But, with such a deterministic approach, the model would produce exactly the same results with repeated stimulations, as it intrinsically incorporates identical initial conditions and identical input dynamics (*σ*) each time. With repeated stimuli, therefore, the deterministic ODE would not be able to produce ‘sensory habituation’ effects without changing the model’s parameters each time. Changing a model’s parameters may produce to some extent the experimental results in ‘sensory habituation’, but underlying biological mechanisms are less obvious. The potential explanations would also be purely dependent on how the model is parameterized. For example, a complicated model requires many free parameters, resulting in a vast pool of alternative parameter combinations to produce similar effects in final model outputs. An equal number of experimental verifications need to be carried out, which is a tedious and difficult task.

Stochastic adaptive sampling from MSCs is a much simpler modelling framework that incorporates relatively small numbers of physiologically relevant parameters. Thus, this current paper shows the power of using this modelling framework as a general approach to model sensory systems. Promising and testable scientific insights from a mechanosensory receptor were produced, in the same way as insights were produced into explaining neuron encoding for fly photoreceptors (Song et al. 2012; Song & Juusola, 2014).

## Conclusions

The aim of the current study was to investigate the common adaptation response profiles seen in photoreceptors and mechanoreceptors. The motivations for conducting such research are: (i) to explore if common adaptive mechanisms could be utilized in neurons from different sensory modalities; and (ii) to derive system level insights into the transduction components in mechanosensory nerve terminals, using understandings derived from the well-studied phototransduction process.

The approach here used computational models to explore how various sensory mechanisms may work together to generate coherent adaptive behaviour at the system level. A general biophysical model was established to map ramp-and-hold stretch stimuli to a mechanosensory primary ending’s receptor current. The model took into account the viscoelastic properties of the accessory receptor muscle fibres and the biophysical properties of the MSCs. A stochastic adaptive sampling framework was adapted from photototransduction to describe how a large population of MSCs collectively produce a complex response pattern to external mechanical stimuli, i.e. ramp-and-hold stretch evokes a large and complex dynamic depolarization during the ramp phase and a smaller plateau depolarization during the hold phase.

The model predicts that the initial dynamic component in a primary sensory ending’s receptor current is likely a combined result of both the mechanical properties of the muscle fibres and the refractory nature of MSCs. Tension increases more rapidly in the ramp phase, resulting in the dynamic component. At the onset of the stretch, all MSCs are sensitive to external stimuli. Later, as they become refractory (inactivated), fewer MSCs are able to respond to the continuous stretch, resulting in a sharp decrease in tension-induced-current after the initial peak response. This induces the initial peak feature in the dynamic component. In a further development of the model, the same mechanism could contribute a faster component in ‘sensory habituation’ of some mechanoreceptors; with repetitive stimuli, a receptor responds less strongly to the subsequent stimulus episodes.

Without any parameter changes, this same model structure for phototransduction also produced the adaptive dynamics in the receptor current in a mechanosensory system. This adaptation emerged naturally out of a population of stochastically operating refractory units (MSCs), just as adaptation emerged from a very large population of refractory microvilli in fly photoreceptors. What is common in the two systems are the assumptions that: (i) there is a huge population of sampling units; (ii) that each sampling unit is refractory in nature; and (iii) that the units operate stochastically according to their own activation and inactivation profiles.

This study is focused on developing the model as a test-bed for scientific insights and practical laboratory evaluations; it provides predictions about adaption dynamics. It suggests alternative candidate mechanisms to explain sensory adaptation in single neurons. It is not our intention to make any claims that these mechanisms are the only ones to explain the peak dynamics in a primary sensory ending’s response. Other mechanisms, such as voltage-gated conductances or Ca^2+^-activated conductances, may provide alternative or contributory effects in various ways for different types of neurons.

## Notes

1 Light intensity changes from a few photons per second in a dark shadow to billions in direct sunlight. Naturally occurring odours used by animals for mate recognition or food identification must be detected at concentrations that differ by several log units. Sound pressure levels range from 1 to 120 dB (7 log units difference), whereas the frequency range for cochlea hair cells of the human ear spans from 20 Hz to 20 kHz. Spindle terminals have to maintain the ability to sense length changes over 3 log units.

2 Reverse engineering, also called back engineering, is the process of extracting knowledge or design information from systems and reproducing it mathematically/materially based on the extracted information. The process often involves disassembling something (a mechanical device, electronic component, computer program, or biological, chemical or organic entity), and analysing its components and workings in detail.
